# Macro‐ and micronutrient effects on phytoplankton in Green Bay, Lake Michigan, and the western basin of Lake Erie

**DOI:** 10.1111/jpy.13519

**Published:** 2024-12-04

**Authors:** Jordyn T. Stoll, James H. Larson, Sean W. Bailey, Christopher B. Blackwood, David M. Costello

**Affiliations:** ^1^ Department of Biological Sciences Kent State University Kent Ohio USA; ^2^ Michigan Trout Unlimited DeWitt Michigan USA; ^3^ Upper Midwest Environmental Sciences Center (UMESC) United States Geological Survey Reston Virginia USA; ^4^ Department of Plant, Soil, and Microbial Sciences Michigan State University East Lansing Michigan USA

**Keywords:** bottle incubation, community composition, Great Lakes, HABs, microcystin, *Microcystis aeruginosa*, micronutrients, nitrogen, phosphorus, phytoplankton

## Abstract

Efforts to reduce the frequency, extent, and toxicity of harmful algal blooms (HABs) require knowledge about drivers of algal growth, toxin production, and shifts in phytoplankton community composition to cyanobacterial dominance. Although labile nitrogen (N) and phosphorus (P) fuel primary production, micronutrients also play roles as the enzymatic engines that facilitate rapid and efficient growth and toxin production. Macro‐ and micronutrient availability can shape community composition and function by selecting for particular taxa. To address how phytoplankton in two Great Lakes subbasins respond to macro‐ and micronutrients, we conducted bottle incubation enrichment experiments using water collected from two blooming and two nonblooming sites in Lakes Erie and Michigan during late summer (August). Three of the four sites exhibited multi‐nutrient limitation of growth. Both blooming sites responded strongest to ${\mathrm{NH}}_{4}^{+}$ enrichment. Both nonblooming sites responded the strongest to ${\mathrm{PO}}_{4}^{3-}$ enrichment, and three of the four sites responded in some way to a mix of micronutrients (Fe, Mn, Mo, Ni, and Zn). *Microcystis aeruginosa* relative abundance increased most with N enrichment, while P enrichment increased the abundance of diatoms and chlorophytes. At the Fox River, N‐enriched communities grew 10%–20% more than non‐N enriched communities (measured as chlorophyll *a*), and N‐enriched communities had, on average, over twice as much microcystin (non‐N communities average MC = 2.45 μg · L^−1^, +N communities MC = 5.35 μg · L^−1^). These overarching trends support the idea that control of HABs may not be effective with a P‐only approach.

**Abbreviations**
ANOVA: analysis of variance
cHABs: cyanobacterial harmful algal blooms
ELISA: enzyme‐linked immunosorbent assay
HABs: harmful algal blooms
ICP‐OES: inductively coupled plasma–optical emission spectrometry
ISO: International Organization for Standards
MC: microcystin
RDA: redundancy analysis

## INTRODUCTION

Harmful algal blooms (HABs; cHABs when composed of cyanobacteria) represent an ecological impairment by altering food webs, restricting recreation, causing benthic anoxia when decomposed, and producing toxins (e.g., microcystins, nodularins, anatoxins, saxitoxins) that are harmful to humans and wildlife (Glibert, 2017; Vasconcelos et al., 2013). As the human population continues to grow, so does the global frequency and extent of HABs (Michalak et al., 2013; Paerl & Barnard, 2020; Paerl & Paul, 2011). During the 1970s and 1980s, the reduction of cultural eutrophication inputs to the Laurentian Great Lakes from point sources decreased the extent of diazotrophic (nitrogen‐fixing or N‐fixing) cHABs (Great Lake Water Quality Board [GLWQB], 1978; Makarewicz, 1993). Although this reduction in point‐source nutrients generally resulted in the oligotrophication of open‐lake areas in the northern Great Lakes (Bunnell et al., 2014; Evans et al., 2011), today blooms of nondiazotrophic cyanobacteria occur in nearshore regions in all five Great Lakes. Notably, the western basin of Lake Erie and Green Bay on the western side of Lake Michigan exhibit prolific annual cHABs composed primarily of *Microcystis aeruginosa* (Berry et al., 2017; Sayers et al., 2016; Stumpf et al., 2012). Extensive work on cHABs has demonstrated that warm temperatures, high macronutrient availability in the epilimnion, and quiescent waters are key drivers of blooms (Gobler et al., 2016; Ho et al., 2019; Jang et al., 2003; Lehman et al., 2013; O'Gorman et al., 2012). Oxidative stress, nutrient form, and herbivory are a few other variables that have been correlated with bloom size and toxicity (Omidi et al., 2018; Zurawell et al., 2005). Future climate trends are expected to further instigate HABs due to increasing temperatures and larger nutrient loads to HAB‐prone areas (Michalak et al., 2013; Sinha et al., 2017; Visser et al., 2016).

Of the known drivers of cHABs, nutrient availability in the epilimnion is most directly related to human activities within the watershed and is the factor that managers can most tangibly control to reduce cHABs. Early studies determined phosphorus (P) had a dominant role in bloom biomass (Schindler et al., 2016; Schindler & Fee, 1974), and springtime P loads are currently used to forecast bloom size in Lake Erie (Stumpf et al., 2012). However, a recent meta‐analysis suggested that high cellular N:P ratios correspond to high concentrations of N‐rich toxin (microcystin) production in cyanobacteria (Brandenburg et al., 2020), with the mechanism(s) associated with stoichiometric imbalance. Another study observed that transcription of the microcystin synthetase gene is regulated by the global prokaryotic N metabolism regulatory protein NtcA, suggesting N availability may be a driver of microcystin production (Ginn et al., 2010). Others have also observed the N control cascade to moderate microcystin production (Beversdorf et al., 2015; Chaffin et al., 2018; Kuniyoshi et al., 2011). Although others have observed opposing trends (Harris et al., 2016; Orihel et al., 2012; Rinta‐Kanto et al., 2009), the literature largely points toward N association with toxin production by *Microsystis aeruginosa* (Hellweger et al., 2022). There is substantial evidence that N (Newell et al., 2019; Paerl et al., 2016; Wagner et al., 2021) and micronutrients (Downs et al., 2008; Havens et al., 2012; North et al., 2007) play larger roles in the size and toxicity of cHABs than previously thought.

In addition to the macronutrients N and P, cyanobacteria require roughly 25 other elements (micronutrients) at lower concentrations for efficient metabolic activity (Kaspari & Powers, 2016). In primary producers broadly, micronutrients serve as cofactors in one‐third of the roughly 4000 enzymes used to facilitate reactions (Kaspari & Powers, 2016), such as zinc (Zn) in the ubiquitous enzyme alkaline phosphatase. Although high concentrations of micronutrients induce oxidative stress, low bioavailable concentrations of micronutrients limit the processes they are involved in. For example, iron (Fe) or molybdenum (Mo) could limit nitrate assimilation by limiting the production of enzymes that reduce nitrate to the usable form ammonia (i.e., nitrate and nitrite reductase; Berges, 1997; Havens et al., 2012; Ivanikova et al., 2007). Micronutrients are not only required for some inorganic macronutrient assimilation but are also needed for efficient recycling of macronutrients from organic molecules (Appendix S1: Table S1 in the Supporting Information), which may be especially important during blooms when inorganic macronutrients are often depleted (Cottingham et al., 2015). Urea, an organic form of N, is the most applied N fertilizer globally (Glibert et al., 2014), and cells hydrolyze urea into ammonia via urease, which requires two nickel (Ni) atoms for each enzyme (Glass et al., 2009). Numerous studies have indicated that urea is a critical N source in bloom‐forming taxa (Belisle et al., 2016; Krausfeldt et al., 2019; Peng et al., 2016), suggesting that Ni bioavailability could be important, especially when reduced inorganic N is in short supply.

Micronutrient bioavailability can not only influence primary producer metabolism, but also shape algal community composition due to the species‐specific and strain‐specific nature of algal nutrient requirements and acquisition physiology (Granéli & Turner, 2006). Lake phytoplankton communities are highly diverse assemblages with members representing deeply divergent evolutionary lines (e.g., diatoms vs. cyanobacteria), making a community‐level response to nutrient enrichment an amalgamation of individual responses by vastly different organisms. This extensive diversity corresponds to a wide array of physically and chemically derived niches in algal communities, which may provide insight into bloom dynamics. For example, the common bloom‐dominating species *Microcystis aeruginosa* is known to utilize urea, potentially giving *M. aeruginosa* a competitive advantage when Ni is not limiting urease production (Belisle et al., 2016). Furthermore, cyanobacteria tend to have greater iron demands than most other primary producers (Brand, 1991). An iron uptake experiment indicated that *M. aeruginosa* iron uptake was independent of cell quotas, and modeling indicated that *M. aeruginosa* was a superior competitor compared to other algae under iron limitation (Nagai et al., 2007). Despite the metabolic and ecological importance of micronutrient availability on phytoplankton processes, little is known about the role of micronutrients in bloom growth or toxicity, as many studies have reported contradictory findings. *Microcystis aeruginosa* growth and toxin production have been positively correlated with Zn and Fe concentrations in laboratory cultures (Li et al., 2009; Zurawell et al., 2005), although another study observed no effect of Zn on toxin concentration (Gouvêa et al., 2008). A recent bottle‐incubation study observed that micronutrient enrichment increased microcystin‐LA concentration in a hypereutrophic reservoir community; however, a lab study on *M. aeruginosa* alone did not indicate micronutrients influenced microcystin production (Wagner et al., 2021). Contradictory findings may be due to the difficulties of working with micronutrients (e.g., contamination, bioavailable forms), differences in responses of various *M. aeruginosa* strains, or differences in *M. aeruginosa* response to enrichment axenically versus when within a wild community. Limitation type is also known to be spatially and temporally dynamic (Bracken et al., 2015; Harpole et al., 2011), making it desirable to measure endpoints other than growth in order to assess the biological mechanism resulting in nutrient limitation or colimitation.

Using a nutrient enrichment experiment, this study aimed to assess the role of ammonium, phosphate, and micronutrients (Fe, Mn, Mo, Ni, and Zn) in bloom community growth, composition, and toxin concentration, with a specific emphasis on the effects of nutrients on cHAB taxa. The endpoints measured allowed assessment of empirical limitation of growth, but the underlying biological mechanism of the nutrient limitation uncovered by these experiments can only be speculated on. Our study established microcosms with field‐collected phytoplankton communities from four locations in Lake Michigan and Lake Erie. We amended the bottle incubations with N, P, and the ionic forms of five micronutrients in a full factorial design under metal‐conscious conditions. We avoided contact of incubation water with metal‐contaminated surfaces by acid washing and triple rinsing with milli‐Q grade water all sample collection and storage vessels and incubation bottles, and we used reagent grade salts for enrichment. By using natural communities and metal‐conscious methods, we aim to eliminate potential experimental flaws that could impact phytoplankton community response to our enrichment scheme. We hypothesized that micronutrients would limit algal growth when ample macronutrients were available (i.e., serial limitation). We predicted that microcystin concentration would increase during incubation in treatments provided with N and micronutrients because microcystin is an N‐rich molecule, production of which may be limited by N availability, and previous studies had indicated that microcystin production may be impacted by Zn and Fe (Li et al., 2009; Zurawell et al., 2005). Finally, we anticipated community composition to change with nutrient treatment, with P additions selecting for fast growing taxa and N additions selecting for nondiazotrophic cyanobacteria. We expected micronutrients to influence whole community composition but without specifying the direction of this change due to the mixture of micronutrients supplied.

## METHODS

### Site info

**FIGURE 1:**
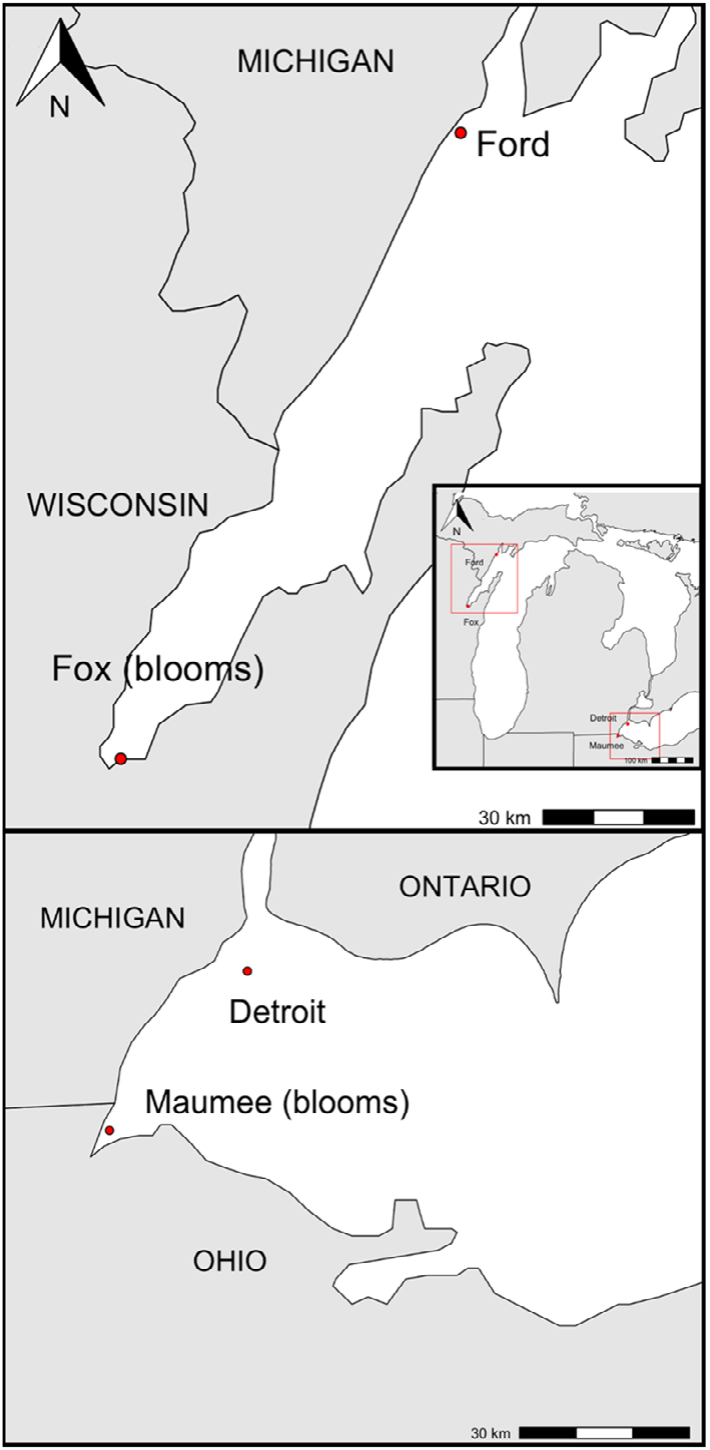
Map of nearshore sites where natural phytoplankton communities were collected. Maumee and the Fox River mouths are eutrophic. The Detroit River mouth is mesotrophic, and the mouth of the Ford River is oligotrophic. [Color figure can be viewed at wileyonlinelibrary.com]

Two sites were selected from each of Lake Erie and Lake Michigan across existing eutrophication gradients in the same basin or embayment (Figure 1). Both the Fox (Lake Michigan) and Maumee Bay (Lake Erie) sites are eutrophic and have annual cHABs of toxin‐producing, nondiazotrophic species complex *Microcystis aeruginosa*, while the Ford River (Lake Michigan) and Detroit River (Lake Erie) sites do not exhibit blooms (Table 1). Maumee Bay is located on the southwest corner of the western basin of Lake Erie and receives water from predominately agricultural watersheds in Ohio and Indiana, resulting in elevated macronutrient conditions. The Detroit River mouth is located south of Detroit and receives advective inputs from the upper Great Lakes. The Fox River discharges eutrophic water at the city of Green Bay into the southern portion of Green Bay. The Ford site was our most oligotrophic site, at the mouth of the Ford River in the northern portion of Green Bay, with a watershed that is largely protected by State Forest lands. At each site, 40 L of whole lake water (passed through a 1 mm screen to remove zooplankton) were collected using a pump 1 m below the surface just prior to peak bloom conditions (August 2017), stored in triple‐rinsed polyethylene containers, and shipped overnight to Kent State University for bottle‐incubation establishment.

**TABLE 1:** Water collection site information and background water chemistry indicate different nutrient conditions across sites.

Site information				Nitrogen (μg N ·L^−1^)			Phosphorus (μg P · L^−1^)		Micronutrients (μg · L^−1^)				
Site	Latitude, longitude	Nutrient status	Chl *a* (μg L)	NH_4_^+^	NO_3_ + NO_2_	TDN	SRP	TDP	Fe	Mn	Mo	Ni	Zn
Maumee Bay	41.70788, −83.45702	Eutrophic	90	29	1668	2245	11	25	25	3	<10	<10	<4
Detroit	41.9639, −83.16373	Mesotrophic	10	76	459	721	2	11	4	<2	<10	<10	<4
Fox	44.54241, −87.99411	Eutrophic	71	58	21	794	9	18	8	3	<10	<10	11
Ford	45.66937, −87.13384	Oligotrophic	1	28	166	450	2	11	39	3	<10	<10	<4

### Incubation

We homogenized and aliquoted whole lake water into thirty‐six 1‐L clear, high‐density polyethylene bottles for each site. We used a full factorial nutrient enrichment design, including three nutrient treatments: elevated N as ammonium (+N; 50 μM; NH_4_Cl), elevated phosphate (+P; 50 μM; KH_2_PO_4_), and a micronutrient mix including Fe, Mn, Zn, Ni, and Mo (+Micro; 3.7, 0.9, 0.08, 0.08, 0.09 μM; FeCl_3_, MnCl_2_, ZnCl_2_, NiCl_2_, Na_2_MoO_4_, respectively) in replicates of four. Micronutrient and P concentrations were based on COMBO medium (Kilham et al., 1998) and were targeted to provide nutrients in excess of biological demand for the incubation timeframe without having toxic effects. Nickle is not included in COMBO medium but was added at the same concentration as Zn to avoid toxic effects, and NH_4_Cl was added at a 1:1 ratio with KH_2_PO_4_. Bottles were incubated at similar temperatures to lake surface conditions at 24 ± 0.6°C for 72 h, which was sufficient time for a growth response while minimizing bottle effects (Lewis & Wurtsbaugh, 2008). Full spectrum grow lights (2000 lumen, SunBlaze #960315) were set to a 16:8 light:dark cycle. Bottles were inverted every 8 h to homogenize the bottle contents, avoid settling, and mimic a natural surface mixing layer. At the same time, bottles were haphazardly reorganized within the incubator to randomize edge effects. Bottles were destructively sampled at the end of the 72‐h incubation.

### Sampling

A subsample from each bottle was allocated to a 20‐mL glass vial and preserved with Lugol's iodine to be assessed microscopically for community composition. Each aliquot was counted to a minimum of 400 natural units via slide transects using an Olympus IX81 inverted microscope with oil immerision at 100x, 400x, or 600x magnification and identified to lowest practical taxonomic unit (typically genus or family; PhycoTech Algae ID workshop materials as primary taxonomic reference). A natural unit is defined as a single cell or a colony of cells. Each colony's total number of cells was quantified, resulting in total cells counted for each sample differing from natural units counted when colonies were present. Samples were cross‐counted among all individuals who counted samples, and no significant sampler bias was identified. A known volume from each bottle (40–150 mL) was filtered onto a glass fiber filter and frozen for chlorophyll *a* analysis. Chlorophyll *a* was measured using an overnight ethanol extraction followed by pigment absorbance reading using a spectrophotometer (Genesys 10S Vis) with an acidification correction to account for spectral interference from pheophytin *a* (Arar, 1997). We collected triplicate filters for analytical replicates of chlorophyll *a* at our two high biomass sites (Maumee and Fox) to ensure large colonies did not skew results.

The four treatment replicates were combined into a composite sample to reduce sample analysis cost and provide sufficient volume, and 150 mL of unfiltered water was frozen and shipped to the United States Geological Survey (USGS) Upper Midwest Environmental Science Center for microcystin measurement. Samples went through three freeze–thaw cycles and were then filtered using 0.45‐μm glass fiber filters. Analytical duplicates were measured for microcystin using microcystins‐ADDA ELISA kits (Abraxis©, Warminster, PA), with a detection limit of 0.1 μg · L^−1^. Samples with concentrations above 2.0 μg · L^−1^ were diluted and reanalyzed to fall within the manufacturer's standard curve. Our microcystin data did not have true replicates due to cost and volume requirements and, thus, did not allow for statistical analysis within sites.

Water filtered through a 0.45‐μm glass fiber filter was preserved with trace metal grade nitric acid to 2% and analyzed for micronutrients using inductively coupled plasma–optical emission spectroscopy (ICP‐OES; Perkin Elmer Optima 8000). Additional filtered water samples were frozen and later analyzed via San++ Skalar nutrient analyzer for total dissolved P (ISO 4‐160), soluble reactive P (ISO 4‐157), nitrate/nitrite (ISO 4‐129), total dissolved N (ISO 4‐135), and ammonia (ISO 4‐118).

### Statistical methods

All analyses were conducted in R (R Core Team, 2018). We have presented chlorophyll *a* results as absolute growth not corrected for initial biomass. Percent abundance of bloom‐forming taxa *Microcystis aeruginosa* was calculated by dividing the number of cells of *M. aeruginosa* by the total number of cells counted in a sample. Differences in chlorophyll *a* and *M. aeruginosa* relative abundances among treatments were assessed using multiway analysis of variance (ANOVA) with separate models for each site. Chlorophyll *a* data from Ford were log‐transformed to account for unequal variance and non‐normality among treatments, but for all other sites, chlorophyll *a* data met ANOVA assumptions. Hill numbers for richness (H0), evenness (HInf), and diversity (H1, H2; Chao et al., 2014; Hill, 1973) were analyzed with ANOVA to determine treatment effect on indices of community diversity. For all ANOVA models, Tukey's honestly significant different post hoc tests were used when *F*‐values were significant to assess pairwise differences in treatment combinations. Community composition data were rarified to the lowest total number of cells counted for each site using the rrarefy function in the vegan package (Oksanen et al., 2018). Community matrices were Hellinger transformed to downweigh the impact of abundant taxa while still permitting Euclidean‐based methods for data analysis (i.e., resulting in analysis of Hellinger distance rather than Euclidean distance; Legendre & Gallagher, 2001). Redundancy analysis (RDA) was conducted for each site to determine the proportion of variation in community composition explained by nutrient treatments and was reported as adjusted *R* ^2^ values. To demonstrate taxa that were best explained by the RDA model at each site, we used the vegan goodness function and then plotted in ordination space the top four to six species scores from RDA1 and RDA2 axes. To demonstrate major changes to community composition by treatment and site, we categorized cell count data into six groups: *M. aeruginosa*, Other Cyanobacteria (no taxa had a greater abundance than 8%), Chlorophytes, Chrysophytes (primarily diatoms, but also golden algae which were <5% of Chrysophyte counts), Other (e.g., dinoflagellates), and Unknown (all taxa <5%). We then averaged treatment replicate values for each group to summarize the results for each site.

## RESULTS

### Broad community responses

**FIGURE 2:**
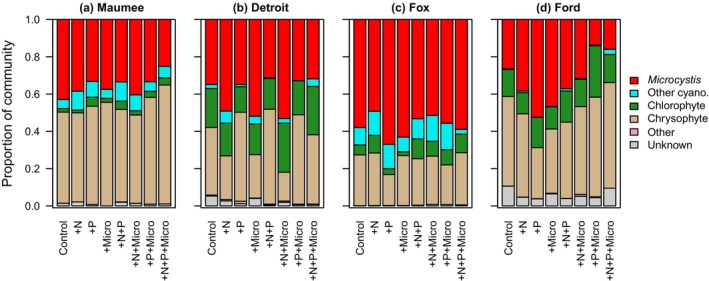
Mean community composition (*n* = 4) per treatment at each site. Cell counts for each replicate were parsed into six categories: *Microcystis aeruginosa*, Other Cyanobacteria, Chlorophytes, Chrysophytes, Other (e.g., dinoflagellates, euglenoids), and Unknown. [Color figure can be viewed at wileyonlinelibrary.com]

As expected, eutrophic sites (Maumee and Fox) had initial communities with greater proportions of *Microcystis aeruginosa* and other cyanobacteria than communities at the meso‐ and oligotrophic sites (Figure 2). Across all sites, *M. aeruginosa* and chrysophytes were the two dominant community members, and other cyanobacteria, chlorophytes, and all other algae combined were less than 30% of the communities. At Maumee, other cyanobacteria primarily consisted of *Pseudanabaena* spp. with relative abundances from 1% to 7% of the community. *Pseudanabaena* spp. was the most common other cyanobacteria at Fox as well, followed by *Dolichospermum* spp.; however, neither genus encompassed more than 5% relative abundance in any treatment community.

Meso‐ and oligotrophic sites were characterized by communities with a greater proportion of chlorophytes than eutrophic sites, although chlorophyte abundance did not exceed more than 30% abundance in any community and did not respond consistently to any nutrient treatment across sites. For all the sites, but especially at Detroit and Ford, there was a trade‐off between chrysophyte and *Microcystis aeruginosa* abundance: as *M. aeruginosa* became more dominant under nutrient‐enriched treatments, chrysophyte abundance decreased. This trend was similar to findings from another nutrient‐enrichment study in which cyanobacteria abundance increased under N‐enriched conditions, with the greatest increased cyanobacteria abundance under N, P, and heightened temperature (Jankowiak et al., 2019).

### Maumee Bay—Eutrophic

**FIGURE 3:**
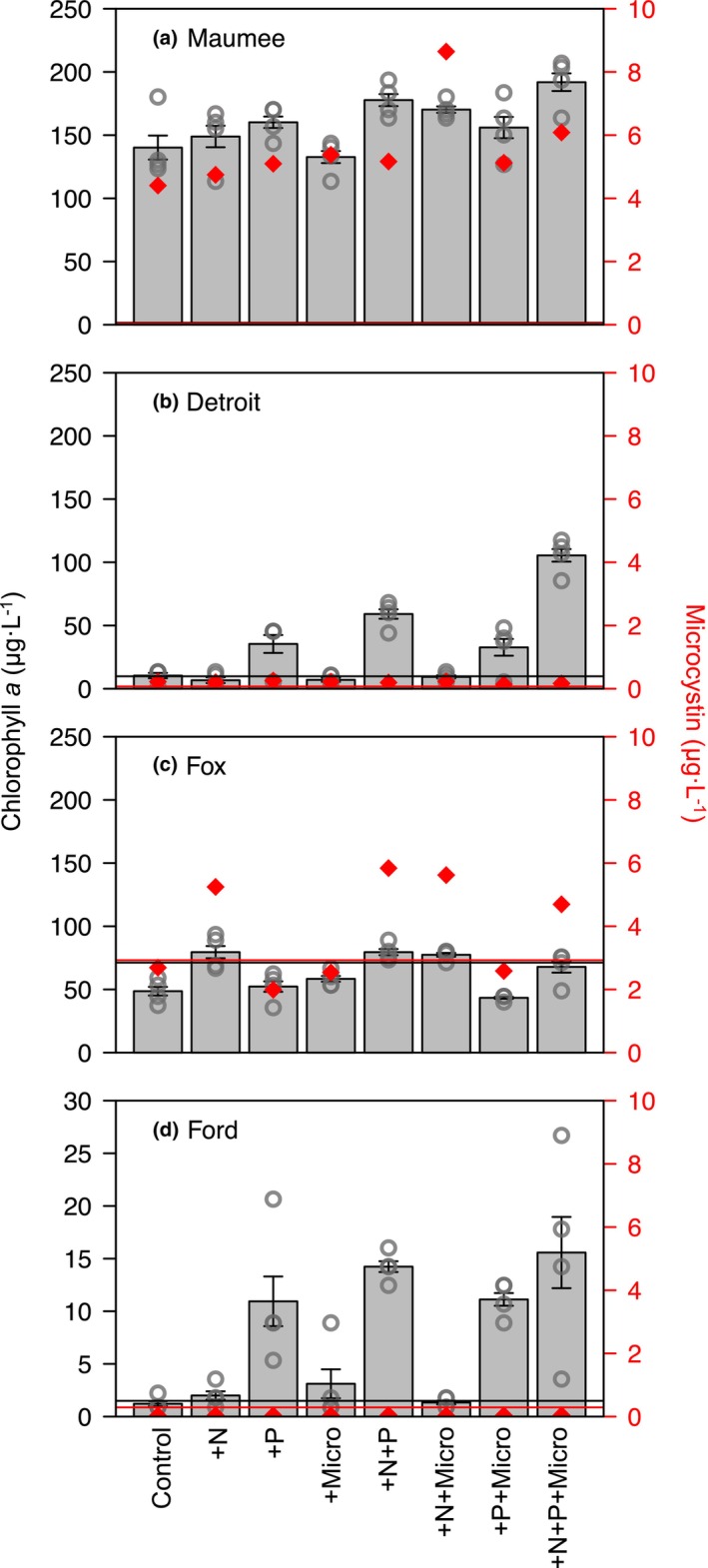
Mean (*n* = 4) chlorophyll *a* is represented as gray bars ±1 standard deviation and each replicate as a dark gray circle (left axis). Microcystin (*n* = 1) concentrations (diamonds) were measured in a composite sample for each treatment (right axis). The initial chlorophyll *a* and microcystin values are indicated by the horizontal black and gray (red online) lines, respectively. Note the change in the chlorophyll *a y*‐axis scale in panel d. [Color figure can be viewed at wileyonlinelibrary.com]

Eutrophic Maumee Bay had the highest initial algal biomass (~90 μg · L^−1^ chlorophyll *a*), which increased further with all nutrient treatments (Figure 3a). Communities enriched with both +N+P and +N+P+Micro grew the most, with 26% and 36% more chlorophyll *a*, respectively, than the control (Appendix S1: Table S2). Multiway ANOVA indicated that single‐nutrient enrichment of +N and +P increased growth, with no significant interactions among treatment combinations (Table 2). The Maumee Bay initial microcystin concentration was 8.6 μg · L^−1^, which was maintained only in the +N+Micro treatment (8.6 μg · L^−1^) and which decreased 29%–44% in all other treatments. Our initial incubation community consisted of 61% *Microcystis aeruginosa* by cell count, and that percentage declined in all treatments (Figure 2a) with the smallest decrease in the control (final abundance = 42% *M. aeruginosa*) and the largest decrease in the +N+P+Micro treatment (final abundance = 26% *M. aeruginosa*) (Table 2). Phosphorus enrichment increased community richness (H0), and micronutrient enrichment decreased richness but increased evenness when in combination with N and/or P (Appendix S1: Figure S1). In the redundancy analysis, adjusted *R* ^2^ indicated 14.3% of the change in community composition was explained by nutrient enrichment type (Table 3). Phytoplankton exposed to +N+P+Micro had distinct community compositions from other treatments (Figure 3a; Appendix S1: Figure S2a), with less *M. aeruginosa* than other enriched communities. The RDA ordination demonstrated that *M. aeruginosa* and *Pseudanabaena* spp. clustered near +N and +Micro enrichment and away from the +N+P+Micro treatment, which agreed with the relative abundance data (Figure 2).

**TABLE 2:** Multiway ANOVA with +N, +P, +Micro, and interactions as independent variables and chlorophyll *a* or percent abundance as dependent variables.

Site analysis	Treatment ANOVA *p*‐value						
	N	P	Micro	N × P	N × MIcro	P × Micro	N × P × Micro
Chlorophyll *a*							
Maumee Bay	**<0.01**	**<0.01**	0.38	0.79	0.09	0.89	0.70
Detroit	**<0.01**	**<0.01**	**0.02**	**<0.01**	**<0.01**	**0.02**	**0.02**
Fox	**<0.01**	0.12	0.34	0.89	0.28	**0.04**	0.49
Ford	0.52	**<0.01**	0.84	0.61	0.20	0.84	0.49
% Abundance *Microcystis aeruginosa*							
Maumee Bay	0.58	**<0.01**	0.36	0.61	0.55	0.50	**0.03**
Detroit	0.52	**<0.01**	0.09	**0.04**	**0.04**	0.26	0.29
Fox	0.34	0.30	0.76	0.47	0.35	0.21	0.46
Ford	0.58	0.09	0.10	**0.05**	0.26	**<0.01**	0.14

*Note*: Chlorophyll *a* data were log‐transformed for the Ford site to correct for nonnormality and unequal variance in the data. The N, P, and Micro columns are main effects, and the four following columns are interactions. Statistical significance (α < 0.05, critical *F*_1,24_ = 4.26) is highlighted in bold.

**TABLE 3:** Results of type I marginal RDA with ANOVA‐like permutation tests for each main effect and treatment interaction.

Site	Full model		Main effects and interactions						
	*F*‐value	Adjusted *R* ^2^	N	P	Micro	N × P	N × Micro	P x Micro	N x P x Micro
Maumee Bay	0.002	14.3%	0.29	**0.04**	**0.01**	0.01	**0.04**	0.17	**0.05**
Detroit	0.001	20.4%	**0.05**	0.07	**<0.01**	**<0.01**	**<0.01**	**0.02**	**0.01**
Fox	0.03	9.8%	0.42	**0.01**	0.16	0.27	0.44	0.09	0.28
Ford	0.001	32.5%	**<0.01**	**<0.01**	**<0.01**	**<0.01**	0.11	**<0.01**	**<0.01**

*Note*: The N, P, and Micro columns are main effects, and the four following columns are interactions. Statistically significant main effects and interactions (α < 0.05) are highlighted in bold.

### Detroit—Mesotrophic

The Detroit River phytoplankton community displayed textbook serial limitation of growth by P, then N, then micronutrients, and growth in the +N+P+Micro treatment was an order of magnitude greater than the control (Figure 3b). The community's growth response to all enrichment types was significant, with +P and +N eliciting stronger effects than +Micro (Table 2). Microcystin concentrations in all treatments and at the start of the experiment were below the detection limit (<0.01 μg · L^−1^). *Microcystis aeruginosa* relative abundance was 20% initially and rose to 30%–50% of total cells during the incubation (Figure 2b). Relative abundance was greatest in the +N, +Micro, and +N+P treatments, while other enriched communities exhibited *M. aeruginosa* abundances similar to the control (Figure 2b). In the redundancy analysis, adjusted *R* ^2^ indicated that nutrient treatments explained 20.4% of the community composition (full model RDA, permutation test, *p* = 0.01), and marginal RDA models indicated that all interactions were significant (Table 3). Communities enriched with +N and +Micro were associated with *M. aeruginosa*, whereas the communities enriched with +P were associated with filamentous and free‐living centric diatoms (Figures 2b and S2b). The +N+P+Micro‐enriched community was composed of a unique assemblage with less *M. aeruginosa* and more diatoms and chlorophytes than other treatment combinations. Although the +N+P+Micro community exhibited the greatest growth, it had the lowest diversity, with all micronutrient‐enriched communities having lower richness (Figure S1).

### Fox—Eutrophic

The eutrophic Fox phytoplankton community was N limited, as all +N‐enriched communities grew 10%–20% more than those not enriched with N (Figure 3c and Table 2). Communities enriched with only +P or +Micro exhibited biomass loss during the incubation period, as did the control, indicating that nutrient enrichment other than +N did not compensate for the strong N limitation in these phytoplankton communities. All communities enriched with +N had microcystin concentrations two to three times the concentration observed in the initial sample and any non‐N‐enriched treatments (Figure 3c, Table S2), with microcystin concentrations on average 2.9 μg · L^−1^ greater in +N‐enriched communities. The percent abundance of *Microcystis aeruginosa* remained above 50% in all treatments and was not affected by nutrient enrichment. Only 9.8% of variation in community composition was explained by the nutrient treatments, indicating that community members other than *M. aeruginosa* were also not strongly influenced by nutrient enrichment (full model RDA, permutation test, *p* = 0.018). *Dolichospermum* spp., a microcystin‐producing cyanobacteria, was in the top five taxa indicated by the goodness scores to influence community composition and was associated with +P, +N +Micro, and +N +P enrichment, although it was not common in the community and likely did not contribute heavily to the total microcystin measured (Figures 2c and S2c). Marginal RDA models indicated that the P main effect was significant (*p* = 0.01), and the RDA ordination demonstrated that +P enriched communities shifted toward *Pediastrum* spp., although this effect was weak, as indicated by the high variability in treatment replicates (Figure S2c). Overall, all diversity indices were ~50% lower in Fox than in other sites, with +N and +N+P having slightly higher diversity than other treatments (Figure S1).

### Ford—Oligotrophic

Growth of the phytoplankton community in the oligotrophic Ford River was P limited (Figure 3d and Table 2), and in all treatments containing P, communities exhibited biomass that was on average 10–13 times higher than the control. All microcystin samples were below the detection limit (<0.01 μg · L^−1^). The N, P, and micronutrients supplied individually increased *Microcystis aeruginosa* relative abundances, and multi‐nutrient enrichment maintained or decreased *M. aeruginosa* relative abundance from control levels (Figure 2d and Table 2). Diversity indices indicated that single‐element additions reduced diversity from the control, but combinations increases diversity. The evenness index (HInf) of the +N+P+Micro community was double that of single‐element‐enriched communities (Figure S1). Nutrient enrichment explained substantial variation in community composition in Ford (full RDA *p* < 0.01, RDA Radjusted *R* ^2^ = 32.7%), and all interactions and main effects were significant other than the N × Micro interaction (Table 3). Concurring with the *M. aeruginosa* relative abundances, the RDA ordination indicated that *M. aeruginosa* was associated with +Micro, +P, and +N single‐nutrient treatments in Ford (Figures 2d and S2d). The +N+P+Micro‐ and +P+Micro‐enriched communities diverged strongly from the other treatments, with the reduction in relative abundances of *M. aeruginosa* in those communities associated with increased abundance of diatoms (e.g., *Cyclotella* spp.) and chlorophytes.

## DISCUSSION

Our study has elucidated the variable individual and combined effects of macro‐ and micronutrients on phytoplankton growth, community composition, and microcystin concentration, within and among locations in Lake Erie and Lake Michigan. Three of our four study sites exhibited multi‐element limitations of growth, altered abundances of toxin‐producing bloom taxa, and unique community compositions for multi‐nutrient enrichment. The only site that exhibited strong P limitation was our most oligotrophic site at the Ford River mouth. Data from these four experiments indicated that nutrients other than P had as much or more impact on growth, toxin concentration, and community composition, concurrent with results from other Lake Erie studies (Baer et al., 2023; Barnard et al., 2021; Chaffin et al., 2022; Hellweger et al., 2022). Although our study only investigated microcystins broadly, Chaffin et al. (2023) observed that microcystin congeners with high N content, such as MC‐RR, were more common under N‐replete conditions in Lake Erie, further supporting that N availability moderates microcystin production, even to the level of microcystin congeners (Chaffin et al., 2023). Although P control continues to be the main management focus around the Great Lakes, our data have indicated that the western basin of Lake Erie and Green Bay algal communities respond to nutrients other than P during bloom times and that by reducing only P and increasing the N:P ratio, toxin‐producing cyanobacteria may be further selected for. *Microcystis aeruginosa* was the primary bloom‐forming and toxin‐producing cyanobacteria throughout this study. *Pseudanabaena* spp. was the second most common cyanobacteria, which has been known to thrive alongside *M. aeruginosa* blooms (Berry et al. 2017; Agha et al., 2016), and no other cyanobacteria had relative abundances above 5% across treatments. Blooming communities, overall, had lower diversity indices compared with nonblooming communities, although this pattern was not consistent for every treatment and was largely impacted by the success of *M. aeruginosa*. Although our experiments were limited to a single point in time (August 2017) and four locations, our results indicate that natural phytoplankton communities may shift dominance to *M. aeruginosa* and produce more microcystin when managed only for P.

During our late‐summer experiment, N and micronutrient enrichment had greater influences on HAB taxa growth and toxin concentration than P enrichment. Maumee Bay and the Fox River, which exhibit annual blooms of *Microcystis aeruginosa*, both started with initial communities composed of ~60% *M. aeruginosa*. However, *M. aeruginosa* relative abundances declined in all incubations of Maumee Bay water, but *M. aeruginosa* remained dominant (i.e., abundance >50%) at the Fox site. These data suggest that N enrichment did not promote the higher abundance of *M. aeruginosa* in the Fox River mouth, yet *M. aeruginosa* produced more toxins under N enrichment. This further suggests that microcystin synthesis was N limited: while N‐enriched communities grew 10%–20% more than non‐N‐enriched communities (measured as chlorophyll *a*), N‐enriched communities produced, on average, over twice as much microcystin (non‐N communities average MC = 2.45 μg · L^−1^, +N communities MC = 5.35 μg · L^−1^). The response we observed to N enrichment in the Fox River mouth is consistent with other studies that have observed that N enrichment facilitates more microcystin production and/or upregulation of microcystin synthesis genes (Brandenburg et al., 2020; Chaffin et al., 2018; Dolman et al., 2012; Giani et al., 2005; Hellweger et al., 2022; Newell et al., 2019; Van De Waal et al., 2009). This trend was not consistent in Maumee Bay, as only the +N+Micro‐enriched community maintained original toxin concentrations; all other treatments decreased microcystin concentrations. Jankowiak et al. (2019) observed a similar trend in microcystin concentrations from a nutrient‐enrichment study in which microcystin concentration was only maintained or slightly increased for N‐containing treatments and decreased for other treatments (Jankowiak et al., 2019).

Detroit River *Microcystis aeruginosa* abundances increased significantly when enriched with N and micronutrients, individually, suggesting that these nutrients facilitate the ability of *M. aeruginosa* to outcompete other algae. Conversely, P enrichment gave the competitive advantage to chlorophytes and diatoms (Figure 2). The Maumee Bay phytoplankton community exhibited a similar response, suggesting that *M. aeruginosa* in the western basin of Lake Erie thrive with N and micronutrient enrichment but are outcompeted by faster growing, eukaryotic taxa when the community is supplied with P. However, our most oligotrophic site at the Ford River mouth in Lake Michigan exhibited a very different response of the phytoplankton community to nutrient enrichment. Growth in Ford was strongly P limited, yet the presence of *M. aeruginosa* in the community increased for single‐nutrient enrichments and declined in multi‐element enrichment combinations. This pattern may indicate that in oligotrophic systems, under strongly stoichiometrically skewed nutrient conditions, *M. aeruginosa* is able to outcompete other taxa and reduce the richness and evenness of the algal community. Other studies have observed that *M. aeruginosa* is a superior competitor under nutrient conditions that stray greatly from the Redfield ratio, especially when reduced N forms are in excess (Chaffin & Bridgeman, 2014; Glibert et al., 2014; Oh et al., 2000), and our data provide further evidence. During the time of our experiments, all four communities were dominated by *M. aeruginosa* and chrysophytes, suggesting that chrysophytes are the strongest competitor with *M. aeruginosa* during late summer. At eutrophic sites, chrysophyte competition with *M. aeruginosa* may be more critical, as Chlorophyte abundance was even further reduced.

Until studies conducted in the 1990s, micronutrients were infrequently considered as limiting nutrients (Martin & Fitzwater, 1988), and even today, they are rarely considered in freshwater systems (Lewis & Wurtsbaugh, 2008). Both Maumee Bay and Detroit River sites had the greatest biomass response in the +N+P+Micro enrichment, which is consistent with serial limitation of micronutrients when sufficient macronutrients are available. Even though micronutrients are not primarily limiting biomass, our study suggests that micronutrients play important roles in shaping phytoplankton community growth and composition in both Lake Erie sites. Our data concur with other results (Chaffin et al., 2018; Newell et al., 2019; Paerl et al., 2016) and suggest that watershed managers may see larger reductions in cHABs if they more holistically manage for all nutrients that influence bloom taxa growth and toxin production, not only for P.

## CONCLUSIONS

Efforts in the Great Lakes to manage HABs are highly focused on reducing nutrient inputs, specifically on reducing P loading (GLWQB, 1978; International Joint Commission, 2014). Our results from Green Bay suggest that when the N:P ratio is skewed from the Redfield ratio by adding N, *Microcystis aeruginosa* is selected for, and microcystin concentration increases. In our study, eutrophic communities were heavily dominated by *M. aeruginosa*, and microcystin concentrations in these communities were stable or increased only in N‐containing treatments, suggesting N, rather than P, drives the production of microcystin. These findings agree with a recent meta‐analysis that observed that N‐rich toxin production corresponds to cellular N:P ratios and nutrient limitation in cyanobacteria (Brandenburg et al., 2020). The implications of focusing efforts on decreasing P loading to the Great Lakes could be examined to inform management decisions, not only in the Great Lakes but also for downstream systems (Paerl et al., 2016). Considering the unique nutrient demands of the microcystin biomolecule, it follows that N enrichment would stimulate higher microcystin concentrations. The +N+Micro enrichment in Maumee Bay was the only treatment to maintain the high initial microcystin concentration, and this result aligns with previous results that have indicated that micronutrients play roles in microcystin production (Li et al., 2009; Wagner et al., 2021). The ecological function of microcystin is still unknown but may vary spatially and temporally across environmental conditions and cyanobacteria taxa (Omidi et al., 2018). At the Fox River mouth, microcystin concentrations correlated with biomass and were heightened in N‐enriched communities, suggesting that microcystin and chlorophyll *a* production were both N limited. Other studies have observed microcystin production commensurate with chlorophyll *a* production (Gouvêa et al., 2008; Long et al., 2001; Lyck, 2004), indicating that microcystin production is indirectly limited by the resource‐limiting light harvesting and, by extension, growth. However, in our study (and as others discuss; Wilhelm et al., 2020), there was not a consistent relationship between cyanobacteria biomass and toxin concentration. Although micronutrients overall had less impact on microcystin concentration than N alone, they may become more important when N limitation is alleviated. This counters the idea that micronutrient demand would increase under severe macronutrient limitation, such as during mid‐bloom, when micronutrients are needed to produce the hydrolysis enzymes that facilitate nutrient regeneration. Conducting enrichment experiments from bloom initiation to senescence may provide more insight into nutrient recycling and the role of micronutrients within blooms.

Our micronutrient treatment consisted of a combination of trace metals (Fe, Mn, Mo, Ni, and Zn) to determine if micronutrients would elicit a response. Our results suggested that these micronutrients do alter community composition, toxin production, and growth (though tertiarily) in Great Lakes phytoplankton. Future studies could investigate individual micronutrients to provide further insight into the specific underlying mechanisms of micronutrient limitation. Determining which micronutrients are limiting can suggest what specific processes are subsequently limited (e.g., Ni limitation and urea decomposition). The freshwater literature would benefit from more micronutrient studies to, at the very least, rule out micronutrient limitations of specific biochemical processes and, at most, discover the importance of micronutrients to planktonic community structure and function and develop novel management tools.

## AUTHOR CONTRIBUTIONS

**Jordyn T. Stoll:** Data curation (equal); formal analysis (equal); investigation (equal); visualization (lead); writing – original draft (lead); writing – review and editing (lead). **James H. Larson:** Conceptualization (equal); data curation (equal); funding acquisition (equal); methodology (equal); resources (equal); writing – review and editing (supporting). **Sean W. Bailey:** Methodology (equal); resources (equal). **Christopher B. Blackwood:** Formal analysis (supporting); methodology (supporting); visualization (supporting); writing – review and editing (supporting). **David M. Costello:** Conceptualization (equal); data curation (equal); formal analysis (equal); funding acquisition (equal); investigation (equal); methodology (equal); project administration (equal); resources (equal); visualization (equal); writing – original draft (equal); writing – review and editing (equal).

## Supporting information


**Table S1.** Micronutrients used in these experiments, their biomolecules, and the biological functions that could be limited by an insufficient supply of the respective element. This table represents well known and common micronutrient mediated functions and is not a comprehensive list.
**Table S2.** Mean chlorophyll *a*, microcystin, and microcystin/chlorophyll *a* ratios (*n* = 4) by treatment and site.
**Figure S1.** Shannon Entropy (H1), Inverse Simpson (H2), Berger‐Parker Evenness (HInf), and Richness (H0) at the lowest practical taxonomic unit (typically genus or family) calculated for each treatment and site. Each Hill index was calculated with 100 permutations on rarified community count data. Error bars are standard errors (*n* = 4).
**Figure S2.** Redundancy analysis (RDA) ordinations for each site. Point color, shape, and fill indicate treatment type (red = N, circle = P, filled = trace). Small points are individual replicates (*n* = 4) and large points are the centroids. Data was rarified and Hellinger transformed prior to RDA analysis. Gray text are taxa that had the highest goodness values for RDA 1 and 2 axes. Statistics for full RDA models and individual treatments are found in Table 3.

## Data Availability

Data associated with this manuscript are available at https://doi.org/10.5066/P14LSD7U (Stoll et al., 2024).
