# Supplementary material for: Macro‐ and micronutrient effects on phytoplankton in Green Bay, Lake Michigan, and the western basin of Lake Erie
Source: J Phycol. 2024 Dec 4;60(6):1514–27. doi: 10.1111/jpy.13519 (PMC11670276; doi:10.1111/jpy.13519)
Supplement: Supplementary file 1 — Table S1. Micronutrients used in these experiments, their biomolecules, and the biological functions that could be limited by an insufficient supply of the respective element. This table represents well known and common micronutrient mediated functions and is not a comprehensive list. Table S2. Mean chlorophyll a, microcystin, and microcystin/chlorophyll a ratios (n = 4) by treatment and site. Figure S1. Shannon Entropy (H1), Inverse Simpson (H2), Berger‐Parker Evenness (HInf), and Richness (H0) at the lowest practical taxonomic unit (typically genus or family) calculated for each treatment and site. Each Hill index was calculated with 100 permutations on rarified community count data. Error bars are standard errors (n = 4). Figure S2. Redundancy analysis (RDA) ordinations for each site. Point color, shape, and fill indicate treatment type (red = N, circle = P, filled = trace). Small points are individual replicates (n = 4) and large points are the centroids. Data was rarified and Hellinger transformed prior to RDA analysis. Gray text are taxa that had the highest goodness values for RDA 1 and 2 axes. Statistics for full RDA models and individual treatments are found in Table 3. [file JPY-60-1514-s001.docx]

Supplemental information for:

**Title**: Macro and micronutrient effects on phytoplankton in Green Bay, Lake Michigan and the western basin of Lake Erie

**Authors**: Jordyn T. Stoll^*^, James H. Larson, Sean W. Bailey, Christopher B. Blackwood, David M. Costello

^*^Corresponding author: Jordyn T. Stoll, Michigan Trout Unlimited, [Jordyn.stoll@michigantu.org](mailto:Jordyn.stoll@michigantu.org)

Contents:

Tables S1–S2

Figures S1–S2

References

**Table S1.** Micronutrients used in these experiments, their biomolecules, and the biological functions that could be limited by an insufficient supply of the respective element. This table represents well known and common micronutrient mediated functions and is not a comprehensive list.

| **Micronutrient** | **Enzyme Cofactors and Biomolecules** | **Functions** |
| --- | --- | --- |
| Manganese (Mn) | Photosystem II, superoxide dismutase, oxalate oxidase | Splitting water molecules in photosynthesis, regulates partitioning of superoxide molecules, oxalate transformation to CO_2_ and H_2_O_2._ (Schmidt & Husted, 2019) |
| Iron (Fe) | Cytochrome, FeS, ferredoxin, NO_3/2_ reduction, nitrogenase | Photosynthesis, respiration, N assimilation and fixation, and cell signaling (Geider & la Roche, 1994) |
| Molybdenum (Mo) | Nitrate reductase, nitrogenase | N assimilation, N fixation (Mendel & Hänsch, 2002) |
| Nickel (Ni) | Urease, hydrogenase | Urea decomposition, hydrogen oxidation associated with N fixation (Muyssen et al., 2004) |
| Zinc (Zn) | Alkaline phosphatase, aminopeptidase, carbonic anhydrase | Cleaves phosphate from organic molecules, decomposes amino acids, carbon transformations (Kaamoush et al., 2022; Sunda & Huntsman, 1995; Vallee & Falchuk, 1993) |

**Table S2.** Mean chlorophyll *a*, microcystin, and microcystin/chlorophyll *a* ratios (*n* = 4) by treatment and site.

| **Site/Nutrient** | **Control** | **N** | **P** | **Micro** | **N+P** | **N+Micro** | **P+Micro** | **N+P+Micro** |
| --- | --- | --- | --- | --- | --- | --- | --- | --- |
| *Chlorophyll a* (ug ⋅ L^−1^) | | | | | | | | |
| Maumee | 140.18 | 148.94 | 160.20 | 132.67 | 177.72 | 170.21 | 156.03 | 191.91 |
| Detroit | 10.35 | 6.68 | 35.38 | 7.01 | 59.07 | 9.35 | 32.71 | 105.47 |
| Fox | 48.62 | 79.54 | 52.29 | 58.41 | 79.54 | 77.43 | 43.39 | 67.86 |
| Ford | 1.22 | 2.00 | 10.95 | 3.12 | 14.24 | 1.34 | 11.13 | 15.58 |
| *Microcystin* (ug ⋅ L^−1^) | | | | | | | | |
| Maumee | 4.41 | 4.75 | 5.10 | 5.38 | 5.17 | 8.65 | 5.13 | 6.09 |
| Detroit | 0.22 | 0.21 | 0.26 | 0.22 | 0.20 | 0.23 | 0.14 | 0.17 |
| Fox | 2.69 | 5.25 | 1.99 | 2.54 | 5.84 | 5.62 | 2.59 | 4.70 |
| Ford | <DL | <DL | <DL | <DL | <DL | <DL | <DL | <DL |
| *Microcystin/Chlorophyll* a *(ug/ug)* | | | | | | | | |
| Maumee | 0.03 | 0.03 | 0.03 | 0.04 | 0.03 | 0.05 | 0.03 | 0.03 |
| Detroit | 0.02 | 0.03 | 0.01 | 0.03 | <0.01 | 0.02 | <0.01 | <0.01 |
| Fox | 0.06 | 0.07 | 0.04 | 0.04 | 0.07 | 0.07 | 0.06 | 0.07 |
| Ford | <DL | <DL | <DL | <DL | <DL | <DL | <DL | <DL |

Micro = Micronutrient mixture of Fe, Mn, Zn, Ni, and Mo, DL = detection limit


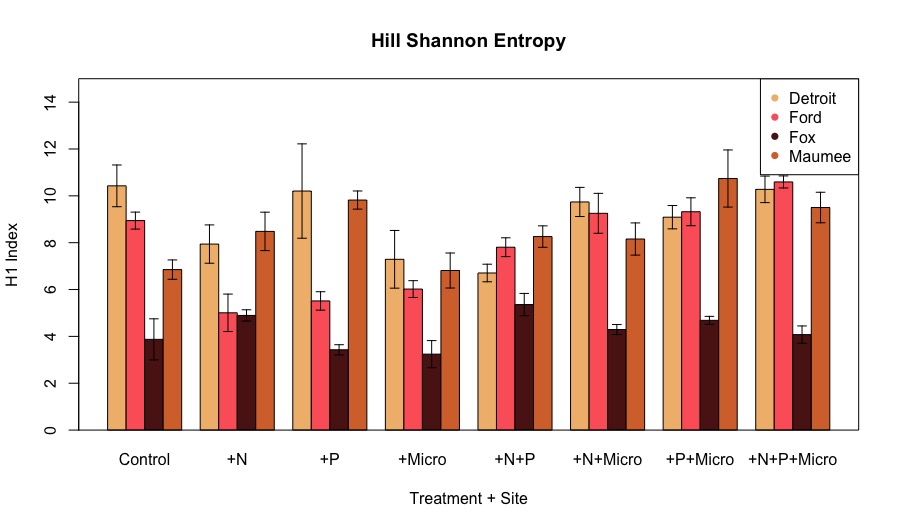


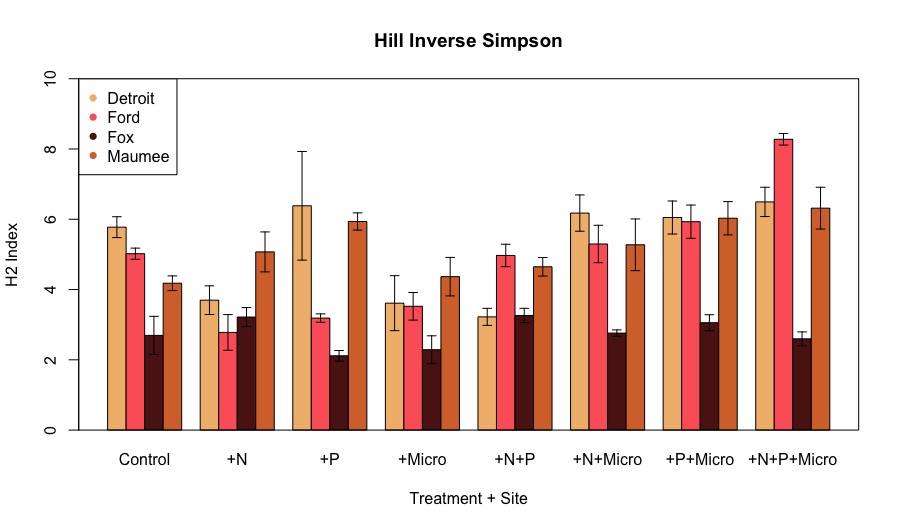


**Figure S1.** (caption below)


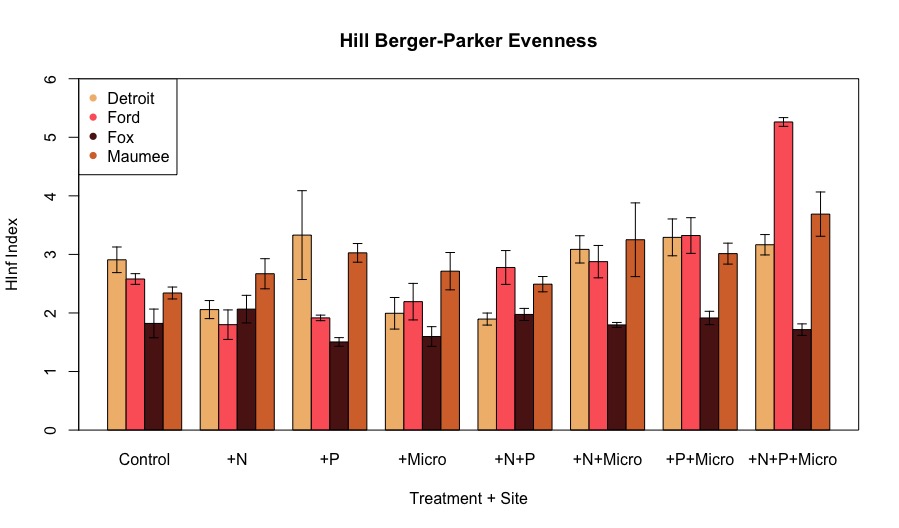


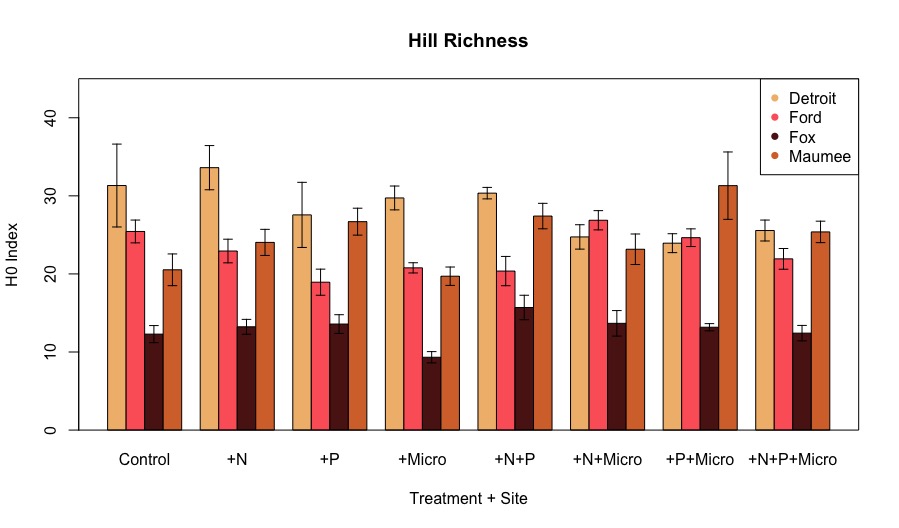
 **Figure S1.** Shannon Entropy (H1), Inverse Simpson (H2), Berger-Parker Evenness (HInf), and Richness (H0) at the lowest practical taxonomic unit (typically genus or family) calculated for each treatment and site. Each Hill index was calculated with 100 permutations on rarified community count data. Error bars are standard errors (*n* = 4).


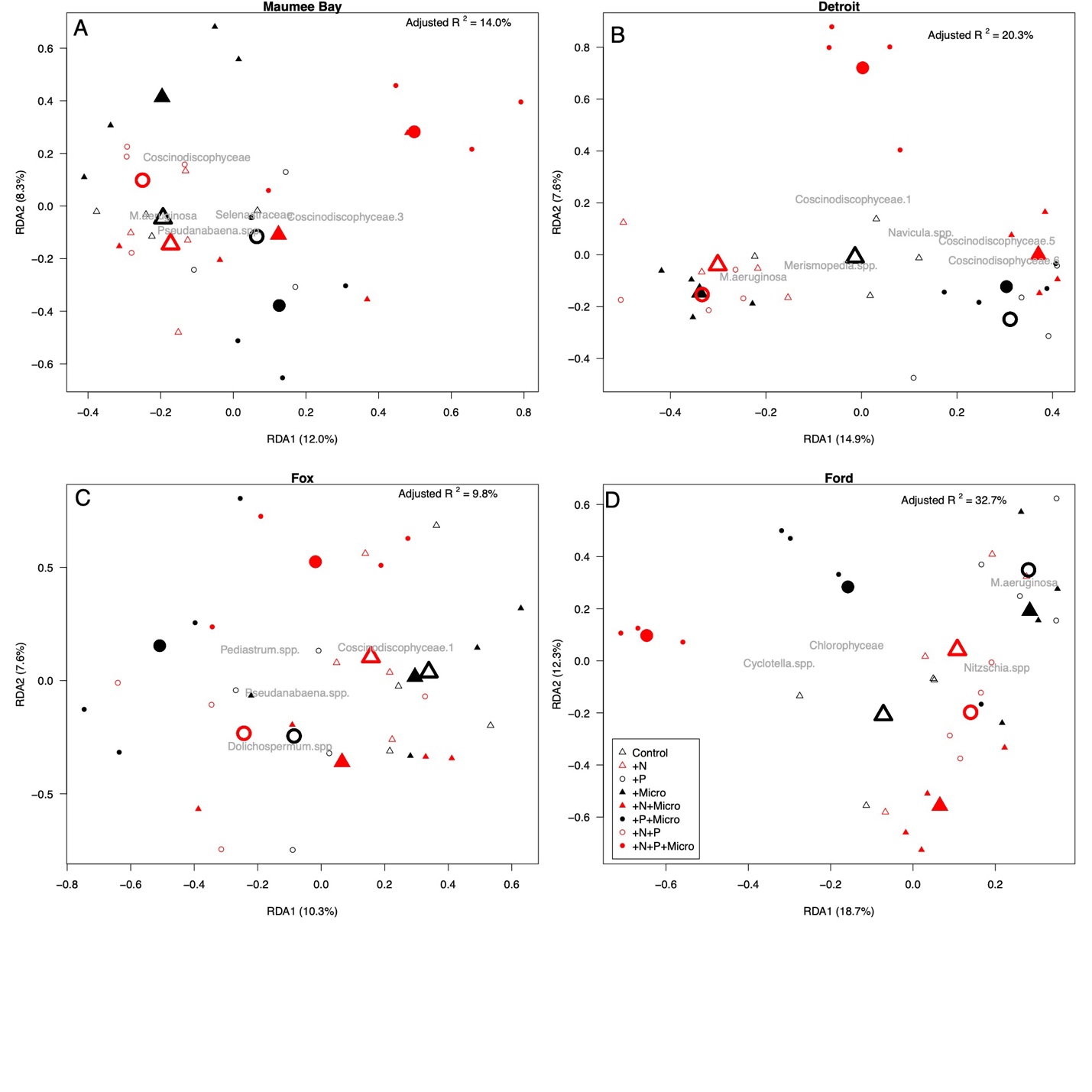


**Figure S2.** Redundancy analysis (RDA) ordinations for each site. Point color, shape, and fill indicate treatment type (red = N, circle = P, filled = trace). Small points are individual replicates (*n* = 4) and large points are the centroids. Data was rarified and Hellinger transformed prior to RDA analysis. Gray text are taxa that had the highest goodness values for RDA 1 and 2 axes. Statistics for full RDA models and individual treatments are found in Table 3.

**References**

Geider, R. J., & la Roche, J. (1994). The role of iron in phytoplankton photosynthesis, and the potential for iron-limitation of primary productivity in the sea. *Photosynthesis Research*, *39*(3), 275–301. https://doi.org/10.1007/BF00014588

Kaamoush, M., El-Agawany, N., Salhin, H. el, & El-Zeiny, A. (2022). Monitoring effect of nickel, copper, and zinc on growth and photosynthetic pigments of *Spirulina platensis* with suitability investigation in Idku Lake. *Environmental Science and Pollution Research*, *29*(52), 78942–78959. https://doi.org/10.1007/s11356-022-21328-1

Mendel, R. R., & Hänsch, R. (2002). Molybdoenzymes and molybdenum cofactor in plants. *Journal of Experimental Botany, 53*(375), 1689–1698. https://doi.org/10.1093/jxb/erf038

Muyssen, B. T. A., Brix, K. v., DeForest, D. K., & Janssen, C. R. (2004). Nickel essentiality and homeostasis in aquatic organisms. *Environmental Reviews*, *12*(2), 113–131. https://doi.org/10.1139/A04-004

Schmidt, S. B., & Husted, S. (2019). The biochemical properties of manganese in plants. *Plants*, *8*(10). https://doi.org/10.3390/plants8100381

Sunda, W. G., & Huntsman, S. A. (1995). Cobalt and zinc interreplacement in marine phytoplankton: Biological and geochemical implications. *Limnology and Oceanography*, *40*(8), 1404–1417. https://doi.org/10.4319/lo.1995.40.8.1404

Vallee, B. L., & Falchuk, K. H. (1993). The biochemical basis of zinc physiology. *Physiological Reviews* *73*(1), 79–118. https://doi.org/10.1152/physrev.1993.73.1.79
